# Thrombocytopenia in *Plasmodium vivax* Malaria: How Significant?

**DOI:** 10.1155/2014/567469

**Published:** 2014-06-17

**Authors:** Arti Muley, Jitendra Lakhani, Saurabh Bhirud, Abhinam Patel

**Affiliations:** SBKS MI & RC, Sumandeep Vidyapeeth, Piparia, Vadodara 390024, India

## Abstract

*Introduction*. Thrombocytopenia is frequently noticed with *P. falciparum* malaria but is less reported and studied with *P. vivax*. *Materials and Methods*. The study was conducted in the Department of Medicine, SBKS MI & RC, Pipariya. We included patients who were diagnosed with vivax malaria. The data regarding their clinical and hematological profile was collected and analysed. *Result*. A total of 66 patients were included. 42 (63%) had platelet count <100000/mm^3^. Mean platelet count was 1,18,650, range being 8000/mm^3^–6,10,000/mm^3^. Amongst those with thrombocytopenia, 16 (38.09%) had anemia, 14 (33.33%) had serum creatinine >1.2 gm/dL, 15 (35.71%) had jaundice (s. bilirubin > 1.2), 2 (4.76%) had altered sensorium, 6 (14.28%) had ARDS, 2 needed ventilator support, and 1 expired. Amongst those with normal platelet count, 5 (20.83%) had anemia and 1 had jaundice whereas none had elevated s. creatinine, altered sensorium, or lung involvement. *Conclusion*. Thrombocytopenia is now being seen more commonly with vivax malaria. Patients with platelet count <1 lac/cumm have more severe disease.

## 1. Introduction

Malaria is a disease of global importance. The World Health Organization (WHO) has reported a worldwide annual incidence of 247 million cases and malarial mortality of one million per year [1].

Infection with* Plasmodium falciparum* (*P*.* falciparum*) is known to cause thrombocytopenia and severe malaria but severe cases in* Plasmodium vivax* (*P. vivax*) malaria patients have been reported in the last decade. With implementation of molecular diagnosis, it became evident that* P*.* vivax *monoinfection could also be involved in multiple organ dysfunction and severe life-threatening disease as seen in* P. falciparum *infection [2, 3]. Many explanations have also been proposed for severe manifestations in vivax malaria like adherence of platelets stimulated by tumor necrosis factor (TNF) to endothelium [4]; bridges formed by platelets between RBCs and endothelial cells as in falciparum malaria [5] and stimulation of platelets by parasitised RBCs triggering apoptosis in endothelial cells pretreated with TNF in a pathway mediated by tumor growth factor- (TGF-) *β*_1_ [6]. Recent evidence showing* P. vivax* infected RBCs adhering to lung endothelial cells and to the placental tissue ex vivo indicates that, in vivax malaria, mechanisms similar to those associated with falciparum malaria severity may be involved [7].

All the complications seen in falciparum malaria are now reported with vivax also [8–10]. A study on pediatric population from Bikaner in northwest India reported a high proportion (63.1%) of severe malaria contributed by* P. vivax* monoinfection. They reported all severe manifestations in vivax malaria in children including severe anemia, thrombocytopenia, cerebral malaria, acute respiratory distress syndrome (ARDS), hepatic dysfunction, renal dysfunction, and abnormal bleeding. They reported thrombocytopenia in 61.5% children and bleeding symptoms in 10.8% cases [9]. A study from Venezuela reported thrombocytopenia in 58.9% children with* P. vivax *malaria, with 25.6% requiring platelet transfusions [11].

Thus, although many studies regarding thrombocytopenia in* P. vivax* malaria are available, most of them were done on pediatric population or included children also. There is lack of studies assessing thrombocytopenia and severity in* P. vivax *exclusively in adults. Hence, we carried out this study to find out the status of thrombocytopenia in* P. vivax* in adult population and find if it has any association with severity.

## 2. Methodology

It was a prospective cohort study. Prior permission for the study was taken from institutional ethical committee. All patients diagnosed with malaria on peripheral smear were enrolled for the study. The patients with falciparum infection either alone or in combination with vivax or who did not give written informed consent were excluded. The patients with underlying disease which may cause similar complications like dengue and sepsis were also excluded.

All patients were subject to the routine laboratory investigations like hematological profile (complete blood count with platelet count), urine routine microscopy, renal function test, serum electrolytes, liver function test, blood sugar levels, and chest X-ray. Viral markers were done in patients with icterus to rule out viral hepatitis. Ultrasonography was also done to study the size and echo texture of the liver and gall bladder, intrahepatic or extrahepatic bile duct dilatation, and signs of portal hypertension. Other special investigations like CT scan of head and arterial blood gas analysis (ABG) were done when indicated. Presenting symptoms and signs were noted along with the information on complications developed.

Thrombocytopenia was defined as platelet count <1 lac/cumm. Anemia was defined as hemoglobin <10 gm/dL. Respiratory distress was defined as oxygen saturation less than 94% at room air or deep breathing (acidotic breathing) or a rapid respiratory rate (>32/min) [12]. The patients were then categorized into two groups according to platelet count (less than 1 lac/cumm and more than 1 lac/cumm) for further analysis. Data was analysed for comparing hematological profile, clinical presentation, complications, and outcome in the two groups.

## 3. Result

A total of 120 patients diagnosed on peripheral smear with malaria were enrolled for the study. 20 were excluded for various reasons (*P*.* falciparum *monoinfection or mixed* P. vivax *and* P. falciparum *infection or coinfection with dengue or associated sepsis). 100 patients had* P*.* vivax *monoinfection as diagnosed on peripheral blood smear. Hence, total 100 patients with* P. vivax* monoinfection were included in the study. The age of included patients ranged from 18 to 50 years. 42 (42%) had platelet count <1 lac/cumm. Average platelet count amongst them was 1,18,651 ± 1,01,937/cumm and mean hemoglobin was 10.69 ± 2.35 mg%.

The patients were divided into two groups based on platelet count (<1 lac/cumm and >1 lac/cumm) for further analysis. Baseline characters were similar amongst the two groups (Table 1).

Gastrointestinal symptoms (nausea, vomiting), shortness of breath, and CNS symptoms (headache, altered sensorium, and loss of consciousness) were more common in <1 lac/cumm group whereas upper respiratory tract symptoms (dry cough, running nose, and sore throat) were more common in >1 lac/cumm group (Table 2). Icterus, tachypnoea, and petechiae were seen in 21.42%, 7.14%, and 2.38% patients, respectively, in the <1 lac/cumm group whereas none was seen in the >1 lac/cumm group.

On lab investigation (Table 3) there was a statistically significant difference in platelet count, serum bilirubin, blood urea, and serum creatinine between the two groups. In the <1 lac/cumm group, 15 (35.7%) had serum creatinine >1.4 mg/dL (OR 8.5), 11 (26.19%) had SGPT >35 IU (OR 6.3), 14 (33.35) had serum bilirubin >1.2 mg/dL (OR 4.28), and 8 (19.04%) had blood urea >40 mg/dL (OR 2.3). These were seen in much less proportion in the other group. Similarly, 7.14%, 11.90%, and 14.28% had ARDS, hepatomegaly, and splenomegaly, respectively, in the <1 lac/cumm group but they were not observed in the other group. Strangely, there was no significant difference in hemoglobin level between the two groups. Odds ratio (OR) of hemoglobin <8 gm/dL between the two groups was 1.14. In <1 lac/cumm group, 2 (4.76%) patients required blood or platelet transfusion and 3 (7.14%) required inotropes and ventilatory support. They were not required in any patient in the >1 lac/cumm group. One patient expired in the <1 lac/cumm group; however, no mortality was seen in the other group. Hemodialysis was not required in any patient in either group (Table 4).

## 4. Discussion

Malaria is a disease that affects almost all blood components. The essential pathological feature of severe falciparum malaria is sequestration of erythrocytes in the deep vascular beds of vital organs leading to cerebral malaria, renal failure, hepatic dysfunction, or ARDS. Hemolysis, reduced cell deformity of parasitized and nonparasitized erythrocytes, increased splenic clearance, reduced platelet production and survival, and increased splenic uptake of platelets cause severe anemia and thrombocytopenia leading to bleeding diathesis.

Initially thrombocytopenia was thought to be a feature of* P. falciparum. *Since the beginning of the 1970s, reports of similar degree of thrombocytopenia in* P. vivax* and* P. falciparum* infections started coming in [12]. Most of the major publications related to frequency of thrombocytopenia in* P. vivax* malaria were published in the late 1990s, probably due to the surge in the availability of automated machines. Around the same time, reports of severe, complicated malaria with* P. vivax* infection also started being published; but regardless of being described as a complication by WHO, thrombocytopenia is not considered to be a severity criterion by itself [13] due to the inability to cause death* per se*.

Many studies have shown a wide range of thrombocytopenia and severity in* P. vivax* malaria but they had distinct selection criteria of the enrolled patients or included only pediatric patients [9, 14]. No study has reported any major bleeding or complication or mortality resulting from even severe thrombocytopenia (platelet count under 50,000/*μ*L) [15, 16]. Only few studies have so far reported mortality from* P. vivax* infection; some have reported multiple complications, but none of these studied adult population exclusively [17–20]. Reports on association of platelet count with peripheral parasitemia have been contradictory. Some studies have reported a negative correlation [21], while many studies in Brazil confirmed a direct positive correlation [15, 16]. Another recent study reported no relation between platelet count and peripheral parasitemia in* P. vivax* monoinfection [22]. The significance of this observation is still unknown.

Although some studies have reported association of thrombocytopenia with severe malaria [23, 24], most of these included pediatric age group also with adults and did not exclude the other endemic causes of thrombocytopenia. In another study, out of the 17 patients affected by any of the WHO malaria severity criteria with confirmed monoinfection, 14 presented with thrombocytopenia, suggesting that this hematological complication can be explored as a marker of severity for* P. vivax* [19]. Tanwar et al. in 2012 reported significant association of severe malaria with platelet count <2 lac/cumm in children [25]. Kochar et al. in 2009 studied adult patients and reported all complications in patients with* Plasmodium vivax* malaria [26].

Thus, there has been a lack of studies on thrombocytopenia in* P. vivax *malaria in adults. Most have included both adult and pediatric populations and have not excluded the other causes of thrombocytopenia. Also, there is no study which has assessed the association of thrombocytopenia with severity in* P. vivax* malaria in adults. Our study included only adult population. We excluded* P. falciparum*, dengue positive as well as sepsis patients as these are the major causes of thrombocytopenia in our region. We also assessed the association of thrombocytopenia with severity in adult* P. vivax* patients. We observed both sequestration-related and nonsequestration-related complications (including abnormal bleeding, cerebral malaria, renal failure, circulatory collapse, ARDS, and jaundice) in our study population. These were significantly more common in patients with platelet count <1 lakh/cumm. ARDS and need of blood or platelet transfusion and inotropes as well as mechanical ventilation were also seen in platelet count <1 lac/cumm group. This group also recorded mortality in one case. Thus, this study highlights the fact that all complications of* P. falciparum* can be seen with* P. vivax* and that the parameter of platelet count <1 lac/cumm may be used as a marker of severity and increased chances of complications. It identifies the set of patients who will need aggressive management.

## 5. Conclusion

The trend of disease with* P. vivax* malaria is changing. Similar incidence of thrombocytopenia is now seen in vivax and falciparum malaria patients. All complications seen in falciparum positive cases are being seen in vivax positive cases also. Thrombocytopenia may not be a cause of mortality by itself, but it can be a marker of increased severity and need of aggressive management. There is a need to reexamine the clinical spectrum and severity of* P. vivax *malaria especially in the light of thrombocytopenia. We also recommend formulation of separate guidelines for management of* P. vivax* with platelet count <1 lac/cumm looking at the severity profile of such patients.

## Figures and Tables

**Table 1:** Basic characteristics of the two groups (<1 lac/cumm and >1 lac/cumm).

	Platelet count	
	<1 lac/cumm	>1 lac/cumm
Age (years)	25 ± 7	27 ± 6 (*P* value 0.13)
Males	26 (61.9%)	37 (63.79%)
Females	16 (38.09%)	21 (36.2%)
RBS (gm%)	115.08 ± 16.77	110.90 ± 21.4 (*P* value 0.38)
BP (systolic, mmHg)	115.90 ± 12.22	118.5 ± 10.09 (*P* value 0.17)

**Table 2:** Frequency of various symptoms and clinical features at presentation in the two groups.

Symptoms	<1 lac/cum	>1 lac/cumm
Headache	21 (50%)	17 (29.16%)
Nausea/vomiting	32 (76.19%)	29 (50%)
Fever	35 (83.33%)	43 (74.13%)
Dry cough	13 (30.95%)	26 (44.83%)
Running nose	7 (16.66%)	16 (27.58%)
Sore throat	8 (19.04%)	19 (32.75%)
Shortness of breath	5 (11.90%)	0
Malaise	5 (11.90%)	8 (13.79%)
Altered sensorium	2 (4.76%)	0
Loss of consciousness	1 (2.38%)	0
Convulsion	0	0
Photophobia	0	0
Pulse	86.09 ± 12.6	80.5 ± 7.15
Pallor	19 (38.09%)	16 (32.75%)
Icterus	9 (21.42%)	0
Tachypnoea	3 (7.14%)	0
Petechiae	1 (2.38%)	0
Cyanosis	0	0
Lymphadenopathy	0	0
Edema	0	0

**Table 3:** Difference in results of investigations between the two groups.

Investigation	<1 lac/cumm	>1 lac/cum	*P* value
Hb (gm%)	10.54 ± 2.26	10.94 ± 2.53	0.54
TLC (per cumm)	6338 ± 3543	6987 ± 3691	0.48
Platelet count (per cumm)	67,619 ± 28,106	2,07,958 ± 121983	<0.05
Blood urea (mg%)	27.78 ± 13.98	22.70 ± 7.49	<0.05
S. creatinine (mg%)	1.01 ± 0.37	0.87 ± 0.2	<0.05
T. bilirubin (mg%)	1.807 ± 1.893	0.941 ± 0.253	0.005
SGPT (IU/L)	33.71 ± 27.92	22.16 ± 5.99	0.013

**Table 4:** Comparison of therapy required in the two groups.

Therapy required	<1 lac/cumm	>1 lac/cumm
Blood transfusion	2 (4.76%)	0
Platelet transfusion	2 (4.76%)	0
Ionotropes	3 (7.14%)	0
Mechanical ventilation	3 (7.14%)	0
Hemodialysis	0	0
Expired	1	0
